# School Mental Health Services: Signpost for Out-of-School Service Utilization in Adolescents with Mental Disorders? A Nationally Representative United States Cohort

**DOI:** 10.1371/journal.pone.0099675

**Published:** 2014-06-09

**Authors:** Marion Tegethoff, Esther Stalujanis, Angelo Belardi, Gunther Meinlschmidt

**Affiliations:** 1 Department of Psychology, Division of Clinical Psychology and Psychiatry, University of Basel, Basel, Switzerland; 2 Department of Psychology, Division of Clinical Psychology and Epidemiology, University of Basel, Basel, Switzerland; 3 Faculty of Medicine, Ruhr-University Bochum, Bochum, Germany; Chiba University Center for Forensic Mental Health, Japan

## Abstract

**Background:**

School mental health services are important contact points for children and adolescents with mental disorders, but their ability to provide comprehensive treatment is limited. The main objective was to estimate in mentally disordered adolescents of a nationally representative United States cohort the role of school mental health services as guide to mental health care in different out-of-school service sectors.

**Methods:**

Analyses are based on weighted data (N = 6483) from the United States National Comorbidity Survey Replication Adolescent Supplement (participants' age: 13–18 years). Lifetime DSM-IV mental disorders were assessed using the fully structured WHO CIDI interview, complemented by parent report. Adolescents and parents provided information on mental health service use across multiple sectors, based on the Service Assessment for Children and Adolescents.

**Results:**

School mental health service use predicted subsequent out-of-school service utilization for mental disorders i) in the medical specialty sector, in adolescents with affective (hazard ratio (HR) = 3.01, confidence interval (CI) = 1.77–5.12), anxiety (HR = 3.87, CI = 1.97–7.64), behavior (HR = 2.49, CI = 1.62–3.82), substance use (HR = 4.12, CI = 1.87–9.04), and eating (HR = 10.72, CI = 2.31–49.70) disorders, and any mental disorder (HR = 2.97, CI = 1.94–4.54), and ii) in other service sectors, in adolescents with anxiety (HR = 3.15, CI = 2.17–4.56), behavior (HR = 1.99, CI = 1.29–3.06), and substance use (HR = 2.48, CI = 1.57–3.94) disorders, and any mental disorder (HR = 2.33, CI = 1.54–3.53), but iii) not in the mental health specialty sector.

**Conclusions:**

Our findings indicate that in the United States, school mental health services may serve as guide to out-of-school service utilization for mental disorders especially in the medical specialty sector across various mental disorders, thereby highlighting the relevance of school mental health services in the trajectory of mental care. In light of the missing link between school mental health services and mental health specialty services, the promotion of a stronger collaboration between these sectors should be considered regarding the potential to improve and guarantee adequate mental care at early life stages.

## Introduction

Mental disorders place a great challenge on the health care system. They are highly prevalent not only in adults [1] but also in adolescents and children [2], [3], [4], [5], [6], [7], with enormous implications for health-related quality of life [8] and health economy [9]. The urgent need to integrate mental health into all aspects of health research and health-care delivery has been realized by major health institutions and scientific journals, as documented for example in The Lancet series on Global Mental Health and strategic research goals and health action plans by the Grand Challenges in Global Mental Health initiative and the American Academy of Pediatrics [10], [11], [12].

Many children and adolescents with mental disorders do not receive adequate care [13], [14], even though effective treatment approaches for mental disorders exist [15]. The median interval between the first onset of a mental disorder and first contact with the treatment sector is nearly a decade [16], although early intervention offers the hope to prevent long-term mental health problems and increase wellbeing and productivity [17].

The school sector has been reported to be a major contact point for children and adolescents with emotional or behavioral problems [13], [18], [19], [20], and the further development of school-based mental health promotion programs for children and adolescents ranks among the top ten challenges identified by the Grand Challenges in Global Mental Health initiative [10]. Results of the International School Psychology Survey reveal that the number of students per school psychologist is alarmingly high, counting from 470 students per school psychologist in Italy to more than 26000 students per school psychologist in parts of Germany [21]. Besides this low ratio of school psychologists to students, the function of school psychologists is rather complex and involves a wide range of tasks among which only one is intervention supply [22], suggesting that sound and comprehensive treatment of children and adolescents with mental disorders remains hardly feasible in the school setting to date. Instead, the school sector has unrivaled access to children and adolescents and, with this unique feature, is exceptionally situated to link children and adolescents with mental disorders to out-of-school services addressing mental health problems and to high-quality care at early disorder stages. However, the role of the school sector as a guide to such out-of-school services remains poorly understood [18], [23]. To improve our understanding of common trajectories of service use in children and adolescents, it has been claimed that it is important to clarify the sequence of health care sector utilization and to move beyond studying single sectors to the study of the potentially more relevant patterns of sector use [13], [24].

The main objective of this study was to estimate in a nationally representative United States cohort, focusing on adolescents with a lifetime mental disorder, the role of school mental health services as guide to care in out-of-school service sectors for the treatment of mental disorders.

## Materials and Methods

### Study sample

This study was conducted using data from the National Comorbidity Survey Replication Adolescent Supplement (NCS-A). The NCS-A, a nationally representative face-to-face survey of 10148 United States adolescents (ages 13–18 years) that was carried out in a dual-frame design between February 2001 and January 2004 [25], [26], [27], has previously been described in detail [25], [26], [28]. Further information on the NCS-A is available at http://www.icpsr.umich.edu/icpsrweb/ICPSR/studies/28581/version/2. Our analyses are based on a subsample of 6483 out of 10123 adolescents who were in school at the time of survey and for whom interviews as well as parent questionnaires (long versions) were available.

### Ethics statement

Adolescents and parents provided written informed consent, and the Human Subjects Committees of both Harvard Medical School and the University of Michigan approved the NCS-A.

### Assessment of mental disorders

To examine lifetime mental disorders in adolescents, trained interviewers used the WHO Composite International Diagnostic Interview (CIDI) Version 3.0, a structured clinical interview assessing major classes of Diagnostic and Statistical Manual of Mental Disorders (DSM)-IV disorders, including affective disorders (i.e. major depressive disorder, dysthymia, bipolar disorder I, bipolar disorder II), anxiety disorders (i.e. agoraphobia, generalized anxiety disorder, social phobia, specific phobia, panic disorder, post-traumatic stress disorder, separation anxiety disorder), behavior disorders (i.e. attention deficit hyperactivity disorder, oppositional defiant disorder, conduct disorder), substance use disorders (i.e. alcohol abuse/dependence, drug abuse/dependence), and eating disorders (i.e. anorexia nervosa, bulimia nervosa, binge eating disorder). The applied version of the CIDI was computer-assisted and has been adjusted for adolescents, with ensured interview quality and concordance with a clinical reappraisal subsample [25], [27], [28], [29]. In addition to the CIDI interview in the adolescents, parents or parent deputy were inquired to respond to a self-administered questionnaire (SAQ) assessing whether their offspring was suffering from attention-deficit/hyperactivity disorder, conduct disorder, oppositional defiant disorder, major depressive disorder, and dysthymic disorder, as for these disorders parental reports have been shown to be of additional diagnostic value [30], [31], [32]. Information from adolescents and parents was integrated whenever it was available from both, and we defined mental disorder as present if DSM-IV criteria were fulfilled either based on adolescent or parent report. Age of onset was specified as the age at which a certain mental disorder developed for the first time. If more than one mental disorder was present within a mental disorder class, age of onset of the first mental disorder in this class was taken as age of onset of this class. Accordingly, if analyses were performed in the subsample of children and adolescents with “any mental disorder”, age of onset of the very first mental disorder was used as age of onset.

### Assessment of service use

Adolescents and parents provided information on child/adolescent mental health service use across multiple sectors, based on the Service Assessment for Children and Adolescents [33]. Levels of agreement between parent and adolescent reports were acceptable [13], [33]. Respondents were asked whether the adolescents ever in their lifetime had gone to see any professionals or facilities for problems with their emotions, behavior, or use of alcohol and drugs, including in- and outpatient care. According to a previous study, service use was classified into the following categories [13]: i) school services, including individual or group psychological counseling/therapy, and ii) out-of-school services, including a) the mental health specialty sector, including community mental health centers or outpatient mental health clinics, mental health professionals, partial hospitalization or day treatment programs, drug or alcohol treatment units/clinics, hospitals and residential treatment centers, b) the medical specialty sector, including emergency rooms, pediatricians or family doctors, and c) other services, including telephone hotlines, self help groups, counselors or family preservation workers, probation or juvenile justice corrections officers or court counselors, spiritual advisors, respite care providers, any other kind of healer, group homes, foster homes, detention centers, prisons/jails, or emergency shelters. Moreover, respondents were asked how old they had been when they first used the reported type of service.

### Statistical analyses

All statistical analyses are based on post-stratification weighted data, which allowed for the correction of minor discrepancies in distributions of school and/or sociodemographic characteristics between the sample and the population [25]. We performed the descriptive analyses of service utilization in different sectors and of the sample's sociodemograhic characteristics by calculating frequencies and percentages of these discrete variables. We computed separate discrete-time proportional hazard models with a non-parametric baseline hazard function, using complementary log-log regression to estimate temporal relationships between school and out-of-school service use for mental disorders, with school service use (versus no school service use) defined as time-varying predictor and mental health specialty service use, medical specialty service use, other service use, and any out-of-school service use defined as outcomes [34]. Analyses were performed in the subsamples of children and adolescents with any i) affective disorder, ii) anxiety disorder, iii) behavior disorder, iv) substance use disorder, v) eating disorder, and vi) mental disorder. We present hazard ratios (HR) and their 95% confidence intervals (CI). To account for the complex structure of the survey data, we used the Taylor series linearization method.

We re-evaluated the results after adjusting *p* values of the 24 main tests for multiple comparisons using the Holm formula [35], [36], [37], thereby accounting for the number of tests.

To validate our results, we conducted the following secondary analyses: To control for potential confounding, we repeated the analyses after adjusting for several sociodemographic factors, as sociodemographic factors have previously been shown to influence mental health service utilization in children and adolescents [13], [38], [39], [40]. We used those sociodemographic factors that have been included in previous epidemiological studies of child and adolescent mental health as predictors of mental disorder prevalence [7], and simultaneously included them in the analyses, with the categories indicated in Table 1.

**Table 1:** Information on mental health service utilization in different sectors and on sociodemographic characteristics of the study sample (N = 3656*).

Service utilization			
**Service sector**		***n***	**Weighted %**
**School mental health service sector**		842	25.24
**Any out-of-school service sector**		1908	55.87
**Mental health specialty sector**		1129	32.70
**Medical specialty sector**		350	10.01
**Other out-of-school service sector**		552	16.73
**Sociodemographic characteristics**			
**Sociodemographic factor**	**Category**	***n***	**Weighted %**
**Sex**	**Female**	**1907**	**49.58**
	Male	1749	50.42
**Age**	13–14 y	1364	34.28
	15–16 y	1428	41.36
	17–18 y	864	24.36
**Race**	Hispanic	475	15.52
	Black	667	15.95
	Other	227	5.10
	White	2287	63.44
**Parental education (highest level of either parent)**	Less than high school	470	14.10
	High school	1092	29.42
	Some college	826	23.22
	College grad	1268	33.27
**Poverty index ratio**	≤1.5	560	15.82
	≤3	711	20.26
	≤6	1209	32.47
	>6	1176	31.46
**Region**	Northeast	674	16.93
	Midwest	1187	23.93
	South	1152	34.28
	West	643	24.86
**Urbanicity**	Metro	1506	46.45
	Other urban	1306	39.31
	Rural	844	14.24
**Number of biological parents living with the adolescent**	0	381	11.03
	1	1517	42.21
	2	1758	46.77
**Birth order**	Oldest	1235	36.71
	Youngest	1047	26.54
	Others	1374	36.75
**Number of siblings**	0	161	4.10
	1	939	25.80
	2	963	27.92
	3 or more	1593	42.19

Abbreviations: y, years.

*Subsample of the National Comorbidity Survey-Adolescent Supplement (NCS-A) including all participants providing self- and parent-reported information on mental disorders, with at least one mental disorder.

Moreover, we repeated the analyses three times, controlling for either i) number of categories in which a mental disorder was present (1/2/> = 3), ii) age of onset of the type of mental disorder characterizing the subsample in which the analysis was performed (affective disorders: 1–10 years/11–12 years/13–14 years/15–18 years; anxiety disorders: 1–4 years/5–6 years/7–10 years/11–18 years; behavior disorders: 1–4 years/5–7 years/8–12 years/13–18 years; substance use disorders: 1–13 years/14 years/15 years/16–18 years; eating disorders: 1–12 years/13 years/14 years/15–18 years; any mental disorder: 1–4 years/5–6 years/7–11 years/12–18 years; limits between the categories were defined by cutoffs as close as possible to the quartiles of the respective age distributions), or iii) presence of further types of mental disorders not characterizing the subsample in which the analysis was performed (yes/no).

We interpreted with caution all results that were statistically significant in the main analyses but not stable after Holm correction or secondary analyses.

There were a low percentage of subjects with missing information on service use and we dealt with missing data by restricting each analysis to subjects with complete data (see Table 2). We evaluated the results using two-sided tests of significance set at a level of 0.05. Statistical analyses were performed using STATA/MP 13 (Stata Corporation, College Station, Texas).

**Table 2:** Discrete-time proportional hazard models for school mental health service utilization (time-varying) predicting out-of-school service use in different sectors.

	Mental health specialty sector			Medical specialty sector			Other out-of-school service sector			Any out-of-school service sector		
	HR	(95% CI)	p-value	HR	(95% CI)	p-value	HR	(95% CI)	p-value	HR	(95% CI)	p-value
**Any affective disorder**	**1.17**	(0.84–1.63)	0.353	**3.01**	(1.77–5.12)	<0.001	**1.67**	(0.95–2.91)	0.071	**1.16**	(0.86–1.57)	0.335
**Any anxiety disorder**	**1.55**	(0.85–2.84)	0.147	**3.87**	(1.97–7.64)	<0.001	**3.15**	(2.17–4.56)	<0.001	**1.99**	(1.41–2.82)	<0.001
**Any behavior disorder**	**1.29**	(0.85–1.95)	0.230	**2.49**	(1.62–3.82)	<0.001	**1.99**	(1.29–3.06)	0.003	**1.50**	(0.92–2.45)	0.103
**Any substance use disorder**	**1.16**	(0.70–1.91)	0.553	**4.12**	(1.87–9.04)	<0.001	**2.48**	(1.57–3.94)	<0.001	**1.47**	(0.92–2.34)	0.102
**Any eating disorder**	**0.19**	(0.02–1.79)	0.144	**10.72**	(2.31–49.70)	0.003	**0.85**	(0.15–4.95)	0.857	**0.48**	(0.13–1.83)	0.276
**Any mental disorder**	**1.76**	(1.11–2.77)	0.017	**2.97**	(1.94–4.54)	<0.001	**2.33**	(1.54–3.53)	<0.001	**1.71**	(1.17–2.49)	0.007

Abbreviations: CI, confidence interval; HR, hazard ratio.

Note: All analyses are based on samples for which information on mental disorders was available from both self and parent report (N = 6483). Due to missing information on service utilization, sizes of the completer samples are as follows: Mental health specialty sector: n = 6358, Medical specialty sector: n = 6326, Other out-of-school service sector: n = 6322, Any out-of-school service sector: n = 6307). To calculate the hazard ratios, periods without any event were dropped.

## Results

### Study cohort descriptives

Out of 6483 adolescents, 3656 adolescents (56.4%) were diagnosed with a lifetime mental disorder. Information on utilization of service sectors addressing mental health problems and on sociodemographic characteristics of the study cohort including these mentally disordered adolescents are presented in Table 1.

### Prediction of out-of-school mental health service utilization by school mental health service use

Table 2 presents results of the discrete-time proportional hazard analyses of the associations between school and subsequent out-of-school service utilization for mental problems in children and adolescents with lifetime mental disorders. School service use predicted subsequent service utilization in the medical specialty sector in children and adolescents with affective disorders, anxiety disorders, behavior disorders, substance use disorders, and eating disorders, and any mental disorder, subsequent service utilization in other service sectors in children and adolescents with anxiety disorders, behavior disorders, and substance use disorders, and any mental disorder, subsequent service utilization in the mental health specialty sector in children and adolescents with any mental disorder, and subsequent service utilization in any out-of-school service sector in children and adolescents with anxiety disorders and any mental disorder.

After adjusting for multiple testing, all but the predictions for subsequent service utilization in the mental health specialty sector and in any out-of-school service sector in children and adolescents with any mental disorder remained statistically significant.

### Secondary analyses

When we repeated the analyses after adjusting for either sociodemographic factors, number of categories in which a mental disorder was present, age of onset of the type of mental disorder characterizing the subsample in which the analysis was performed, or further types of mental disorders not characterizing the subsample in which the analysis was performed, levels of significance were mostly comparable to those presented in Table 2. However, the temporal prediction of mental health specialty service use by antecedent school service use was no longer significant in the subsample of children and adolescents with any mental disorder in the secondary analyses adjusting for sociodemographic factors (HR = 1.36, 95% CI = 0.85–2.19, *p* = 0.20), and number of disorder categories (HR = 1.50, 95% CI = 0.94–2.39, *p* = 0.08), and the temporal prediction of any out-of-school service use by antecedent school service use was no longer significant in the subsample of children and adolescents with any mental disorder in the secondary analyses adjusting for sociodemographic factors (HR = 1.48, 95% CI = 0.99–2.22, *p* = 0.06). Instead, the temporal prediction of other service use by antecedent school service use reached marginal significance in the subsample of children and adolescents with any affective disorder in the secondary analyses adjusting for number of disorder categories (HR = 1.81, 95% CI = 1.04–3.13, *p* = 0.04) and further types of mental disorder (HR = 1.79, 95% CI = 1.05–3.04, *p* = 0.03). Detailed results of the secondary analyses are available on request.

## Discussion

This study, conducted with 6483 adolescents of a nationally representative United States cohort, provides as yet missing association estimates of school and subsequent out-of-school service utilization for mental problems in children and adolescents with a lifetime mental disorder. Results indicate that school services may serve as guide to certain out-of-school service sectors addressing mental disorders, especially the medical specialty sector, but not the mental health specialty sector.

Our results complement previous descriptive evidence regarding service use patterns in adolescents with mental disorders [13], [18], [23] by elucidating the role of the school service sector in the trajectory of child and adolescent service utilization for mental disorders, and suggest that the school sector plays a central role as guide to the medical specialty sector.

The roles of the medical and the mental health specialty sectors in mental health care have been addressed previously. Several studies have confirmed the prominent position of the medical specialty sector in providing services for mental disorders [41], even though the mental health specialty sector has been reported to be the major service sector for children and adolescents with mental disorders [13] and the most common subsequent service sector in adolescents entering the service system through the school mental health sector [18]. While mental health care in the medical specialty sector has improved over the past decade through a better understanding of mental disorders, the availability of practicable screening tools, provision of psychotherapy in these settings, and further development and promotion of psychotropic medications that are mostly prescribed by primary care physicians [24], [42], [43], [44], most primary care pediatricians and child and adolescent psychiatrists believe that pediatricians should identify and refer, but not treat, their patients' mental health problems [45]. Indeed, the appropriateness of treatment of mental disorders in the medical specialty sector as compared to the mental specialty sector remains a matter of debate [46], [47], [48], [49], [50], which should be kept in mind when evaluating the referral patterns observed in the present study.

There is considerable evidence that sociodemographic characteristics of children and adolescents with mental disorders influence whether or not services for mental disorders are utilized; such sociodemographic characteristics include, amongst others, ethnicity, family income, different patient, parental and familial factors, health insurance coverage, and service availability [13], [38], [39], [40], [51], [52], [53]. Much less is known about what influences, in those making use of mental health care, the sequence of sector utilization on their pathway through the service system; for example what influences whether children/adolescents with mental disorders are seeking care in the medical sector or the mental health sector. Therefore, it has been claimed that it is important to clarify the role of potential factors, including referral patterns by professionals, service availability, child and family preferences, professional competencies, and financial considerations, in order to better understand what determines the service paths of children and adolescents seeking help for mental disorders [13].

Our study has several strengths, including the large nationally representative sample [25], a fully structured diagnostic interview covering a wide range of adolescent mental disorders, with good quality criteria [25], [29], and the integration of adolescent and parent information on adolescent mental disorders, as previously suggested [32]. Given the good response rate and the small amount of missing data, it is unlikely that loss of subjects has introduced relevant selection bias.

The study also has several limitations to be considered, including amongst others the cross-sectional design of the study, as previously discussed [28], [54].

Moreover, first, service utilization has been assessed by self-report, but the validity and reliability of such data has been challenged, especially in terms of non-response and recall bias, which is no issue in large health care utilization databases [55], [56]. However, the NCS-A has not been linked with health care utilization databases, but instead provides self-reported information on service utilization for mental disorders based on a well-established instrument [13], [33]. In adults, concordance between self-reported health care and mental health care utilization and registration data has been shown to be fair [57], [58], [59], and the rather low magnitude of bias in estimates of health care utilization due to non-response in health surveys has previously encouraged the continued use of interview health surveys [60]. A study on the reliability of children's and adolescents' responses on the Child and Adolescent Services Assessment, the groundwork of the Service Assessment for Children and Adolescents [33], revealed that reports of lifetime service use were as reliable as were reports of service use in the preceding three months, even though it should be acknowledged that respondents reported inpatient, other overnight and juvenile justice services more reliably than outpatient and school services [61]. It should also be noted that relying on health care utilization databases faces other limitations, including the exclusion of services provided outside the health care system [56], and the advantages of checklists over routine data sources have been previously documented [62].

Second, even though we controlled for several potential confounders, we cannot exclude confounding by unconsidered factors, including disorder severity. However, we did not reveal a relationship between school mental health service use and subsequent utilization of the mental health specialty sector, and controlling for number of disorder categories did not change the results remarkably, both of which suggests that disorder severity did not confound the presented results in a relevant manner.

Third, the observed associations do not inform about causal but about temporal relationships, and even though the study of causality is wanted, it remains challenging in such a large representative cohort. Until such studies are available, the elucidation of the position of the school sector in the trajectory of service use for mental disorders allows for a better understanding of common service paths of children and adolescents, as has previously been claimed [13].

Fourth, the aim of our analyses was to estimate whether school mental health service use serves as a guide to the use of any out-of-school service sectors; however, we did not compare school service use with use of a specific out-of-school service sector regarding their prediction of service use in further sectors. Therefore, it may be interesting to scrutinize in future studies whether the here observed referral patterns are specific for school mental health services.

The clinical and public health relevance of the role of the school sector in the trajectory of service use for mental disorders in children and adolescents becomes evident against the background of the urgent need to reduce unmet mental disorder treatment demands and improve access to mental health care [13], [16], together with the limited ability of school mental health services to provide comprehensive treatment on-site on the one hand and their unique position in accessing children and adolescents with mental disorders and linking them to out-of-school services on the other hand [21], [63]. It has previously been criticized that the coordination of the different health care sectors is often insufficient, thus representing a barrier for the patients and impairing the supply with appropriate treatments [64]. By raising the awareness of unidirectional referral patterns of school mental health services, our results may pave the way to improve the school mental health service system and multilateral collaborations between sectors where required, which is in line with current strategic research goals and task forces [10], [63]. Notably, our findings raise the question as to whether the school mental health sector sufficiently takes advantage of its unique position in linking mentally disordered students with the out-of-school service system beyond the medical specialty sector.

The supply of mental health services in places where children and adolescents are easily accessible is only one among several action steps that have been proposed to increase access to mental health care for children and adolescents with mental disorders. Further suggestions include the improvement of access to information on available mental health care options, inclusion of children and adolescents in treatment planning, and offering assistance in finding one's way through the rather complicated service system [65]. The bottom line is that, to be most effective in increasing access to child mental health care, strategies should target not only at structural barriers to care, such as inadequate insurance coverage, but also at barriers related to the understanding of mental health problems and services; for example, public education campaigns may help to increase the awareness and knowledge of mental disorders and mental health care services [66], [67].

Whether the here presented role of school mental health services as a guide to out-of-school services for mental disorders in the United States is generalizable to other countries remains to be elucidated, as not only the school mental health system but also insurance and health care systems, including access to mental health care systems, vary considerably worldwide [64], [68].

Future studies should focus on the role of school mental health services in the trajectory of mental health service use in other countries. Moreover, future research should include prospective data and the integration of self-report of service utilization and service utilization databases. It will also be important to evaluate the appropriateness of the mental health service use trajectory emanating from the school mental health sector and to learn about modifiable factors influencing referral patterns of school mental health services, in order to be able to take action and guide or optimize such referral patterns, if necessary.

To the best of our knowledge, this is the first comprehensive study of the role of the school mental health sector as a guide to mental health care in out-of-school sectors, using data of mentally disordered adolescents of a nationally representative United States cohort. Results indicate that school mental health services may serve as a guide to certain out-of-school service sectors for children and adolescents with various mental disorders, especially the medical specialty sector, but not the mental health specialty sector. While highlighting the relevance of school mental health services in the trajectory of mental care, our findings also suggest to further investigate into a stronger collaboration between school mental health services and mental health specialty services in order to improve and guarantee adequate mental care at early life stages.
